# TMEM158 expression is negatively regulated by AR signaling and associated with favorite survival outcomes in prostate cancers

**DOI:** 10.3389/fonc.2022.1023455

**Published:** 2022-11-01

**Authors:** Jian Huang, Wang Liu, Da Zhang, Biyun Lin, Benyi Li

**Affiliations:** ^1^ Center for Pathological Diagnosis and Research, The Affiliated Hospital of Guangdong Medical University, Zhanjiang, China; ^2^ Department of Urology, The University of Kansas Medical Center, Kansas City, KS, United States; ^3^ Department of Pathology and Laboratory Medicine, The University of Kansas Medical Center, Kansas City, KS, United States

**Keywords:** TMEM158, prostate cancer, patient survival, disease progression, AR signaling, immune filtration

## Abstract

**Background:**

Membrane protein TMEM158 was initially reported as a Ras-induced gene during senescence and has been implicated as either an oncogenic factor or tumor suppressor, depending on tumor types. It is unknown if TMEM158 expression is altered in prostate cancers.

**Methods:**

Multiple public gene expression datasets from RNA-seq and cDNA microarray assays were utilized to analyze candidate gene expression profiles. TMEM158 protein expression was assessed using an immunohistochemistry approach on a tissue section array from benign and malignant prostate tissues. Comparisons of gene expression profiles were conducted using the bioinformatics software R package.

**Results:**

COX regression-based screening identified the membrane protein TMEM158 gene as negatively associated with disease-specific and progression-free survival in prostate cancer patients. Gene expression at the mRNA and protein levels revealed that TMEM158 expression was significantly reduced in malignant tissues compared to benign compartments. Meanwhile, TMEM158 downregulation was strongly correlated with advanced clinicopathological features, including late-stage diseases, lymph node invasion, higher PSA levels, residual tumors after surgery, and adverse Gleason scores. In castration-resistant prostate cancers, TMEM158 expression was negatively correlated with AR signaling activity but positively correlated with neuroendocrinal progression index. Consistently, in cell culture models, androgen treatment reduced TMEM158 expression, while androgen deprivation led to upregulation of TMEM158 expression. Correlation analysis showed a tight correlation of TMEM158 expression with the level of R-Ras gene expression, which was also significantly downregulated in prostate cancers. Tumor immune infiltration profiling analysis discovered a strong association of TMEM158 expression with NK cell and Mast cell enrichment.

**Conclusion:**

The membrane protein TMEM158 is significantly downregulated in prostate cancer and is tightly associated with disease progression, anti-tumor immune infiltration, and patient survival outcome.

## Introduction

The transmembrane protein (TMEM) family is a group of proteins that anchor on the biological membrane and span the entire lipid bilayers (1). Although their functions are mostly unknown, several members of TMEM family proteins were reported as either oncogenic or tumor suppressors in human cancers (1–3).

TMEM158 was initially reported as a receptor for brain injury-derived neurotrophic peptide (BINP), involved in neuronal cell survival (4). Meanwhile, TMEM158 expression was upregulated exclusively during Ras/ETS2-mediated senescence (5). In recent years, TMEM158 upregulation was reported in multiple human cancers, including colon cancers (6), thyroid cancers (7, 8), triple-negative breast cancers (9), pancreatic cancers (10), laryngeal cancer (11), ovarian cancers (12), and glioblastomas (13). TMEM158 oncogenic action was demonstrated in cell culture and mouse xenograft models derived from colon cancer (6), pancreatic cancer (10), ovarian cancer (12), glioblastoma (13), and triple-negative breast cancer (14). In contrast, TMEM158 gene silencing rendered cisplatin resistance in lung cancer cells (15). However, there is no report about TMEM158 expression in prostate cancer yet in the literature.

Prostate cancer is the most diagnosed cancer and the second leading cause of cancer-related death in American men (16, 17). Patients with localized prostate cancers usually have a favorite 5-year survival outcome (about 99%) after surgery; however, the 5-year survival rate drops drastically to only 30% for patients with distal metastatic cancers (16). Treatment for metastatic prostate cancers starts with androgen deprivation therapy since the prostate organ and cancers are androgen responsive. Unfortunately, this therapy only lasts a short period, and all patients eventually progress to the castration-resistant (CRPC) stage (18). Prostate cancers with castration resistance are a critical obstacle in the clinic due to no means to cure, and about 10-17% of CRPC patients even developed a more aggressive neuroendocrinal differentiation (so-called NEPC) (19). For NEPC patients, only platinum-based chemotherapy is available with substantial side effects and a short response duration (19). Therefore, there is an urgent need to develop clinically reliable biomarkers for disease prognosis and therapeutic targeting.

We conducted a transcriptome-wide prognostic factor screening in this study using the TCGA-PRAD RNA-seq dataset. COX aggression-based statistical analysis identified 716 protein-coding genes significantly associated with disease-specific survival and progression-free interval. Among these genes, six membrane-anchoring proteins from the transmembrane protein TMEM family were noticed and further analyzed, yielding the TMEM158 expression as a potent prognostic factor. Our in-depth analysis revealed that TMEM158 expression at the mRNA and protein levels was significantly reduced in malignant tissues compared to benign tissues. TMEM158 downregulation was significantly associated with advanced clinicopathological features, including late-stage tumors, lymph node invasion, residual tumors after surgery, high PSA levels, and high Gleason scores. In CRPC patients, TMEM158 expression was negatively correlated with the AR activity score but positively with the NE feature score. In prostate cancer cells, TMEM158 expression was suppressed after androgen addition but increased after androgen removal. In addition, TMEM158 expression was strongly correlated with R-Ras but not K-Ras or N-Ras gene expression and anti-tumor immune infiltration. Our results suggest that TMEM158 expression is a potent prognostic factor for disease progression and survival outcomes and that TMEM158 might serve as a potential therapeutic biomarker for prostate cancer treatment.

## Materials and methods

### Gene expression analysis

TMEM158 gene expression at the mRNA level was analyzed using the RNA-seq dataset obtained from the Cancer Genome Atlas Prostate Adenocarcinoma project (TCGA-PRAD). In this project, there were 52 cases with matched normal-tumor specimens, plus 447 patients with only tumor specimens at the time of analysis. The RNA-seq data at the level 3 HTSeq-FPKM (Fragments Per Kilobase per Million) format were downloaded from the TCGA portal (https://portal.gdc.cancer.gov). They were then converted to TPM (transcripts per million reads) format (log_2_ [TPM + 1]) for analysis. All comparisons were conducted at the web-based XIATAO bioinformatics platform (www.xiantao.love).

Additional TMEM158 gene expression profiles were obtained from the following datasets: 282 early-onset prostate cancer patients from the DKFZ group (20), 119 patients with metastatic prostate cancer from the MCTP group (21), as well as 126 prostate cancers (22) and 12 prostate cancer organoids (23) from the MSKCC group. The RNA-seq data for gene expression analysis in castration-resistant prostate cancers were obtained from the SU2C/PCF project, which contains 444 specimens from 429 patients (24). These datasets were downloaded from the cBioportal platform (www.cbioportal.org).

### Patient survival outcome assessments

The association of gene expression with patient survival outcomes was assessed using the Kaplan-Meier curve approach. Patients were stratified into high and low groups of the candidate gene expression using the minimum p-value strategy (25). The hazard ratio (HR) was statistically analyzed using the Log-rank test to determine the significance.

### Immunohistochemistry analysis

The tissue array slide for immunohistochemistry (IHC) analysis was commercially obtained from Novus Biologicals, LLC (Avenue, CO). The array contained nine case-matched normal and tumor sections, as well other 31 unmatched tumor sections from primary prostate cancer patients. Two cases had a Gleason sum score of 6 or 10, 15 cases for Gleason 7 or 9, and 6 cases for Gleason 8. The anti-TMEM158 polyclonal antibody was IHC-validated by the Human Protein Atlas project (HPA074974) and was obtained from Signa-Aldrich (St Louis, MO). The immunosignal was visualized using the VECTASTAIN Elite kit (catalog #PK8200) from Vector Labs (Burlingame, CA). The positive immunosignals were analyzed with a semi-quantitative approach, as described in our previous publication (26).

### TMEM158 expression in prostate cancer cells

TMEM158 expression profiles in LNCaP cells after androgen stimulation or deprivation were analyzed using the public datasets (NCBI GDS2057 and GDS3358), available at the NCBI GEO portal. For androgen stimulation experiments, prostate cancer LNCaP cells were cultured in 8% charcoal-stripped fetal bovine serum (cFBS) for three days and then treated with dihydrotestosterone (DHT, 10 nM) for 24 h. Total cellular RNAs were extracted with the OligoTex kit (QIAGEN, Valencia, CA). Gene expression profiles were examined using the Affymetrix Human Genome U133AB platform (27). Androgen deprivation experiment was conducted on LNCaP cells using 10% cFBS (testosterone < 0.03 ng/ml in cFBS) for up to 5 months. Total cellular RNAs were extracted with Qiagen RNeasy Mini Kit (Qiagen, San Diego, CA). Gene expression profiling experiments were performed on the Affymetrix Human Genome U133 Plus 2.0 arrays (Affymetrix, Santa Clara, CA), as described in a previous publication (28).

TMEM158 expression in PC-3 cell-derived xenografts from nude mice with high or low metastatic potentials was analyzed using the NCBI GDS2865 dataset. Total RNAs were extracted from flash-frozen subcutaneous xenograft tumors using the RNeasy Midi kit (Qiagen, Valencia, CA). Hybridization was conducted on the human U133A chips (Affymetrix, Santa Clara, CA), as described in the previous publication (29).

TMEM158 expression at the protein levels was analyzed using western blot assays. LNCaP cells were seeded in 10% fetal bovine serum (FBS) and then were moved to hormone-depleted condition with 2% charcoal-stripped FBS with or without anti-AR drugs Enzalutamide and Abiraterone to mimic the androgen deprivation therapy in the clinic. Cells were harvested 24 h later, and total cellular proteins were extracted using RIPA buffer. An equal amount of proteins was subjected to SDS-PAGE electrophoresis, and the proteins on the PVDF membrane were probed with an anti-TMEM158 antibody (HPA074974, Signa-Aldrich Co., St Louis, MO). Immunosignals were developed using the ECL agent obtained from Santa Cruz Biotech (Santa Cruz, CA), as described in our publication (30).

### Immune infiltration profiling in prostate cancers

The Spearman correlation analysis between candidate genes and 24 types of tumor immune infiltration profiles was conducted using the gene set variation analysis (GSVA) approach (31, 32).

### Statistical analysis

RNA-seq data for gene expression at the mRNA level were presented as Log_2_ [TPM + 1]) value. Data in each group were also shown with the MEAN plus/minus the SEM (standard error of the mean). ANOVA test with multiple comparisons or two-group Student *t*-test were conducted to determine the significance of the differences. Data analysis and visualization were primarily performed using the R packages (version 3.6.3) and GraphPad software (version 9.1.0)

## Results

### TMEM158 gene expression is downregulated in prostate cancer

We conducted a COX regression analysis with the TCGA-PRAD RNA-seq dataset to screen prognostic factors for patient survival outcomes. Patients were stratified into the high or low expression groups at the median value of gene expression level. Our analysis revealed that 1159 genes were significantly (p < 0.05) correlated with disease-specific survival (DSS, **Supplemental Table S1**) and 9026 genes with progression-free intervals (PFI, **Supplemental Table S2**). There were 716 common genes in both DSS and PFI lists (**Supplemental Table S3**). Among these 716 genes, six were from the transmembrane protein family, TMEM67, TMEM106C, TMEM126B, TMEM158, TMEM225B, and TMEM272.

**Figure 1:**
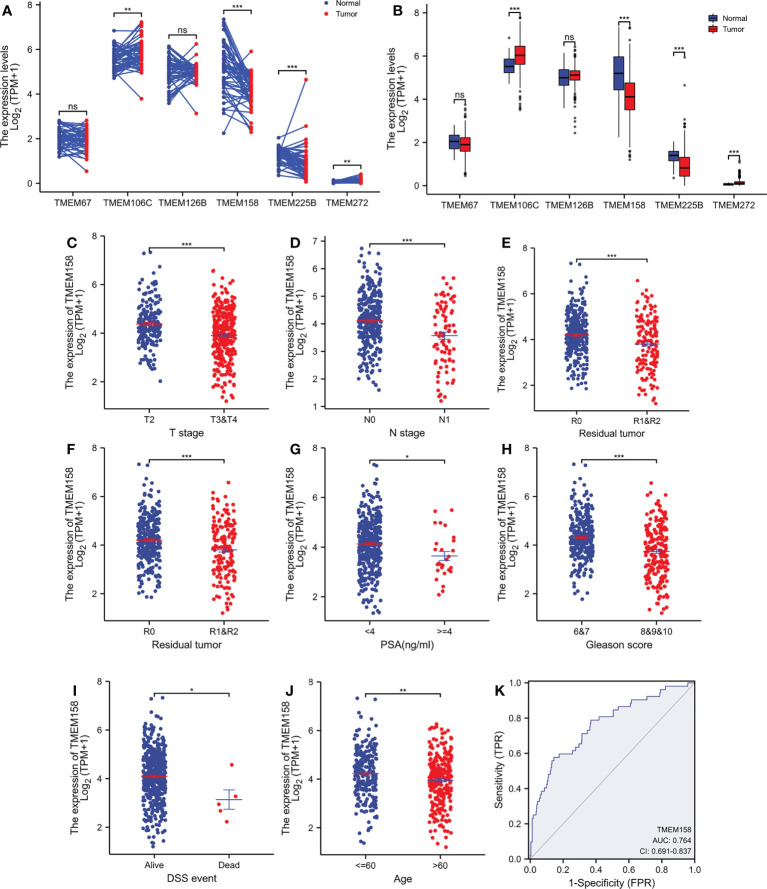
TMEM158 expression is downregulated in prostate cancers. **(A–B)** Gene expression at the mRNA levels was compared between normal and tumor tissues using the TCGA-PRAD RNA-seq dataset with a case-matched pair-wise approach (panel **A**, n = 52, paired *t*-test, **p < 0.01, ***p < 0.001) and group cohort approach (panel **B**, normal n = 52; tumor = 499, Wilcoxon rank sum test, ***p < 0.001). **(C–J)** TMEM158 expression was compared between different patient groups stratified by clinicopathological categories. The asterisks indicate a significant difference (Wilcoxon rank sum test, *p < 0.05; **p < 0.01, ***p < 0.001). **(K)** ROC analysis was conducted for TMEM158 expression in distinguishing normal and tumor tissues.

We compared the expression levels of these six TMEM genes in case-matched benign and malignant tissue pairs. As shown in **Figure 1A**, TMEM67 and TMEM126B did not significantly differ in gene expression levels between benign and malignant tissues. TMEM158 gene expression was significantly lower in malignant tissues than in benign compartments, except for 4 (7.7%) cases. The other three genes (TMEM106C, TMEM225B, and TMEM272) were significantly higher in malignant tissues. Similar results were obtained when group comparisons were used to compare gene expression levels of these six genes in benign and malignant tissues (**Figure 1B**).

We then conducted a qualitative comparison of gene expression to analyze its correlation with multiple clinicopathological features. Based on the median value, patients were stratified into low and high gene expression groups (TMEM158^low^ and TMEM158^high^). Our analysis revealed that there were significantly more patients in the TMEM158^low^ group than the TMEM158^high^ group associated with late-stage tumor, lymph node invasion, older age (> 60), higher PSA level (≥ 4 ng/ml), and higher Gleason scores (> 8) (**Table 1**), indicating a substantial impact on disease progression. In contrast, patients with higher expression of TMEM106C and TMEM225B were only associated with late T-stage, and higher Gleason score tumors (**Supplemental Tables S4** and **S5**), indicating a less impact on disease progression. In addition, higher TMEM272 expressions were more often observed in the black race group (**Supplemental Table S6**), indicating a potential race disparity.

**Table 1:** TMEM158 expression and clinicopathological parameters in prostate cancer patients.

Characteristic	Low expression of TMEM158	High expression of TMEM158	p	statistic	method
n	249	250			
T stage, n (%)			< 0.001	16.08	Chisq.test
T2	73 (14.8%)	116 (23.6%)			
T3	166 (33.7%)	126 (25.6%)			
T4	7 (1.4%)	4 (0.8%)			
N stage, n (%)			0.042	4.13	Chisq.test
N0	173 (40.6%)	174 (40.8%)			
N1	50 (11.7%)	29 (6.8%)			
M stage, n (%)			0.618		Fisher.test
M0	223 (48.7%)	232 (50.7%)			
M1	2 (0.4%)	1 (0.2%)			
Primary therapy outcome, n (%)			0.236	4.25	Chisq.test
PD	17 (3.9%)	11 (2.5%)			
SD	14 (3.2%)	15 (3.4%)			
PR	24 (5.5%)	16 (3.7%)			
CR	159 (36.3%)	182 (41.6%)			
Race, n (%)			0.185	3.37	Chisq.test
Asian	6 (1.2%)	6 (1.2%)			
Black or African American	35 (7.2%)	22 (4.5%)			
White	201 (41.5%)	214 (44.2%)			
Age, n (%)			0.042	4.12	Chisq.test
<=60	100 (20%)	124 (24.8%)			
>60	149 (29.9%)	126 (25.3%)			
Residual tumor, n (%)			0.099		Fisher.test
R0	147 (31.4%)	168 (35.9%)			
R1	85 (18.2%)	63 (13.5%)			
R2	3 (0.6%)	2 (0.4%)			
Zone of origin, n (%)			0.425		Fisher.test
Central Zone	1 (0.4%)	3 (1.1%)			
Overlapping / Multiple Zones	68 (24.7%)	58 (21.1%)			
Peripheral Zone	71 (25.8%)	66 (24%)			
Transition Zone	6 (2.2%)	2 (0.7%)			
PSA(ng/ml), n (%)			0.042	4.14	Chisq.test
<4	200 (45.2%)	215 (48.6%)			
>=4	19 (4.3%)	8 (1.8%)			
Gleason score, n (%)			< 0.001	22.44	Chisq.test
6	16 (3.2%)	30 (6%)			
7	105 (21%)	142 (28.5%)			
8	38 (7.6%)	26 (5.2%)			
9	87 (17.4%)	51 (10.2%)			
10	3 (0.6%)	1 (0.2%)			
Age, median (IQR)	62 (56, 66)	61 (56, 66)	0.163	33370	Wilcoxon

To verify this association of TMEM158 downregulation with disease progression, we performed a quantitative analysis on TMEM158 gene expression with clinicopathological features. Similar to the qualitative results shown in **Table 1**, TMEM158 downregulation was significantly associated with multiple adverse clinicopathological features, including late-stage tumors, lymph node invasion, residual tumors after surgery, high PSA (> 4 ng/ml), higher Gleason scores (> 8), short disease-specific survival, and older age (**Figures 1C–J**). ROC analysis indicated that TMEM158 expression levels exhibited a strong predictive potential (AUC = 0.764) for distinguishing malignant from benign prostate tissues (**Figure 1K**). These data suggest that TMEM158 expression is downregulated in prostate cancer tissues and is associated with disease aggressiveness.

**Figure 2:**
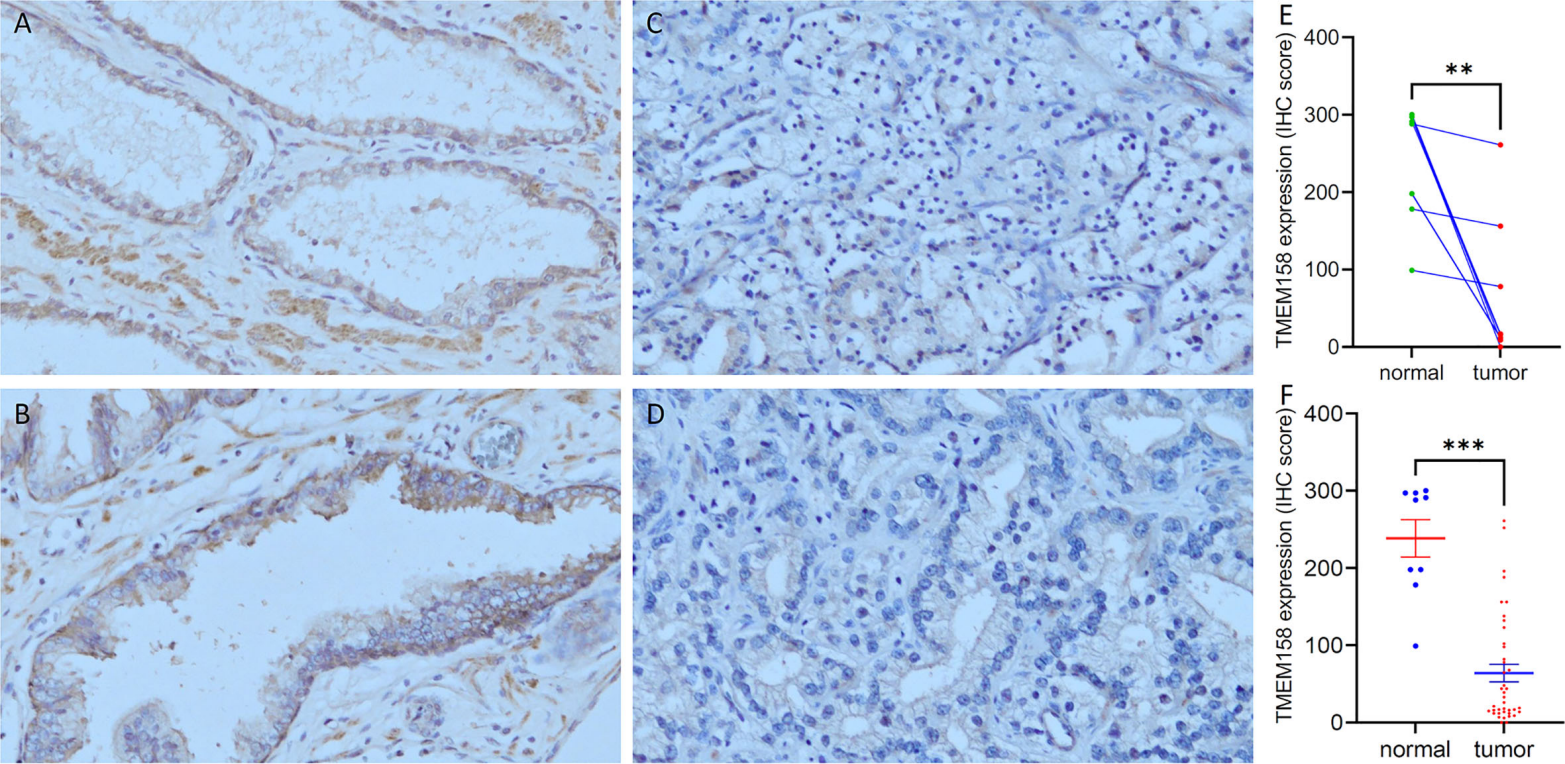
TMEM158 protein expression is decreased in prostate cancer tissues. TMEM158 protein expression was examined in prostate cancer tissues with an immunohistochemistry approach, as described in our recent publication (30). Representative microscopic images were taken from benign prostatic tissues **(A–B)** and malignant prostate tissues **(C–D)**. Magnification x 200. Semi-quantitative data (MEAN and SEM) of the immunosignals were shown for the pairwise comparison **(E)** and group cohort comparison **(F)**. The asterisk indicates a significant difference (Student *t*-test, **p < 0.001, ***p < 0.0001).

To confirm TMEM158 downregulation in prostate cancer tissues, we conducted an immunohistochemical study on a set of prostate tissue arrays containing nine benign and 40 malignant tissue sections. As shown in **Figures 2A, B**, benign prostate epithelial cells exerted a strong immunosignal, mainly around the cellular membrane location, with significantly less signal in the nuclear compartment. Conversely, malignant prostate tissues mostly showed a negative result or weak immunosignal in the cytoplasm compartment (**Figures 2C, D**). Semi-quantitative data analysis demonstrated a significantly lower level of TMEM158 expression in tumor tissues compared to benign tissues, either in case-matched pairwise comparison (**Figure 2E**) or group cohort comparison (**Figure 2F**). These data demonstrated that TMEM158 expression is significantly downregulated in prostate cancer.

**Figure 3:**
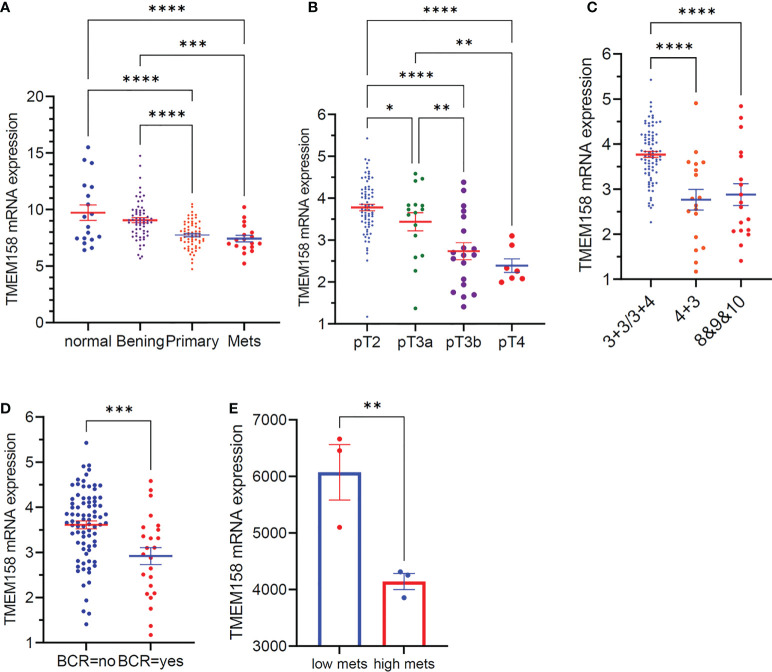
TMEM158 downregulation is associated with disease progression in prostate cancers. **(A)** TMEM158 expression was analyzed using the Affymetrix Human Genome U95 Version 2 Array. Data were extracted from NCBI GDS2545 (33, 34). Case number: normal = 18, benign = 63, primary tumor = 65, metastasis = 18. **(B–D)** TMEM158 expression RNA-seq data were extracted from a cohort of 292 early-onset prostate cancer patients on the cBioportal platform (20). Case number: pT2 = 74, pT3a = 16, pT3b = 19, pT4 = 7; Gleason score category 3 + 3/3+4 = 82, 4 + 3 = 18, 8and 9and 10 = 18; BCR-no = 81, BCR-yes = 24. **(E)** TMEM158 expression data were extracted from NCBI GDS2865 in PC-3 cell xenografts with low or high metastatic potential (29). Gene expression data were obtained using Affymetrix Human Genome U133A Array (n = 3). The asterisks indicate a significant difference (Wilcoxon rank sum test or Student t-test, *p < 0.05; **p < 0.01, ***p < 0.001, ****p < 0.0001).

We also validated the results of TMEM158 downregulation from the TCGA-PRAD RNA-seq dataset with additional gene expression datasets (33). As shown in **Figure 3A**, TMEM158 expression gradually decreased from normal prostate and benign hyperplasia to localized primary and metastatic cancer tissues. In a cohort of early-onset prostate cancer patients (20), TMEM158 expression levels were shown at a significantly lower level in late-stage tumors (**Figure 3B**), higher Gleason scores tumors (**Figure 3C**), and biochemically relapsed (BCR) cancers (**Figure 3D**). Consistently, in prostate cancer PC-3 subline cells with different metastatic potential (29), TMEM158 expression was significantly lower in highly metastatic than weakly metastatic subline cells (**Figure 3E**). These data validated that TMEM158 expression is downregulated in prostate cancers and is tightly associated with disease progression.

### TMEM158 expression is negatively modulated by AR signaling

**Figure 4:**
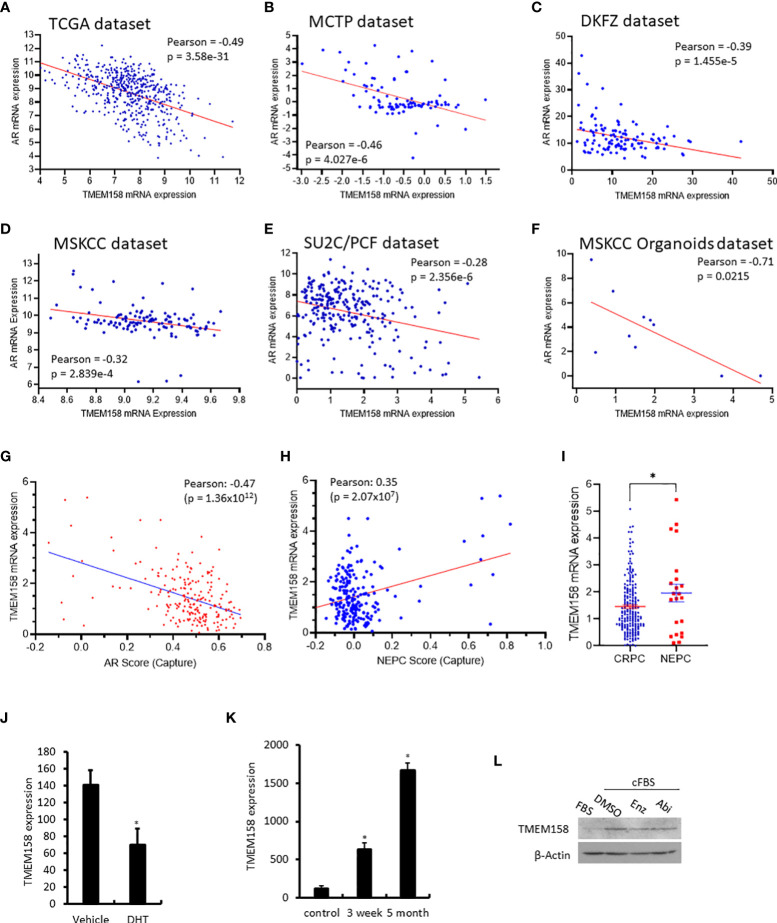
TMEM158 downregulation is associated with AR signaling activity in CRPC cancers. **(A–F)** Pearson correlation analysis was conducted between the expression levels of AR and TMEM158 genes using the RNA-seq data from previously published datasets, as indicated in the figures. **(G–I)** Spearman correlation analysis of TMEM158 expression with the AR activity score **(G)** and NEPC score **(H)**, as well as the group comparison of TMEM158 expression between CRPC and NEPC tumors **(I)**, were conducted using the RNA-seq dataset (24) obtained from 429 patients with metastatic castration-resistant prostate cancers (Student t-test, *p < 0.05). **(J–K)** TMEM158 expression cDNA microarray datasets were extracted from NCBI GDS2057 **(J)** (27) and GDS3358 **(K)** (28). Briefly, LNCaP cells were treated with dihydrotestosterone (DHT, 10 nM) or kept in androgen-depleted media for 3-week or 5-month. Total RNAs were extracted for gene expression analysis using the Affymetrix Human Genome U133 Plus 2.0 Array. The asterisk indicates a significant difference from the vehicle or control culture condition (Student *t*-test, *p < 0.05). **(L)** LNCaP cells were exponentially grown in culture media supplied with 10% FBS or in 2% charcoal-stripped FBS. Cells were also treated with anti-AR agents Enzalutamide (Enz, 10 μM) or Abiraterone (Abi, 10 μM) in 2% charcoal-stripped FBS for 24 h. Cellular proteins were used for the anti-TMEM158 immunoblot assay. Action blot served as a protein loading control.

Since the AR signaling pathway is critical in prostate cancer development and progression (18), we analyzed the correlation of TMEM158 expression and AR gene expression in six RNA-seq datasets generated from prostate cancer tissues with different stages. As shown in **Figure 4**, TMEM158 expression had a significantly negative correlation with AR expression in all six datasets. The Pearson r values ranged from -0.71 to -0.32 in cancers at the androgen-responsive stage. The Pearson r value was -0.28 but still had a strong significance (p = 2.356e-6) in castration-resistant cancers. Further analysis of the CRPC dataset (24) revealed a significant negative correlation between TMEM158 expression and the AR activity score (**Figure 4G**), a signature index of AR signaling activity in CRPC patients (35).

In contrast, TMEM158 expression was positively correlated with the NEPC score (**Figure 4H**), a gene expression signature of neuroendocrinal progression after AR antagonist treatment (35), and TMEM158 expression levels in NEPC tumors were significantly higher than in CRPC tumors (**Figure 4I**). Experimentally, androgen treatment reduced TMEM158 expression (**Figure 4J**), while androgen depravation in the culture media led to increased levels of TMEM158 expression in prostate cancer LNCaP cells (**Figure 4K**). Androgen deprivation also increased TMEM158 protein levels in prostate cancer LNCaP cells (**Figure 4L**). These results strongly suggest that the AR signal pathway negatively modulates TMEM158 expression in prostate cancers.

### TMEM158 upregulation is a robust prognostic factor for a favorite survival outcome

**Figure 5:**
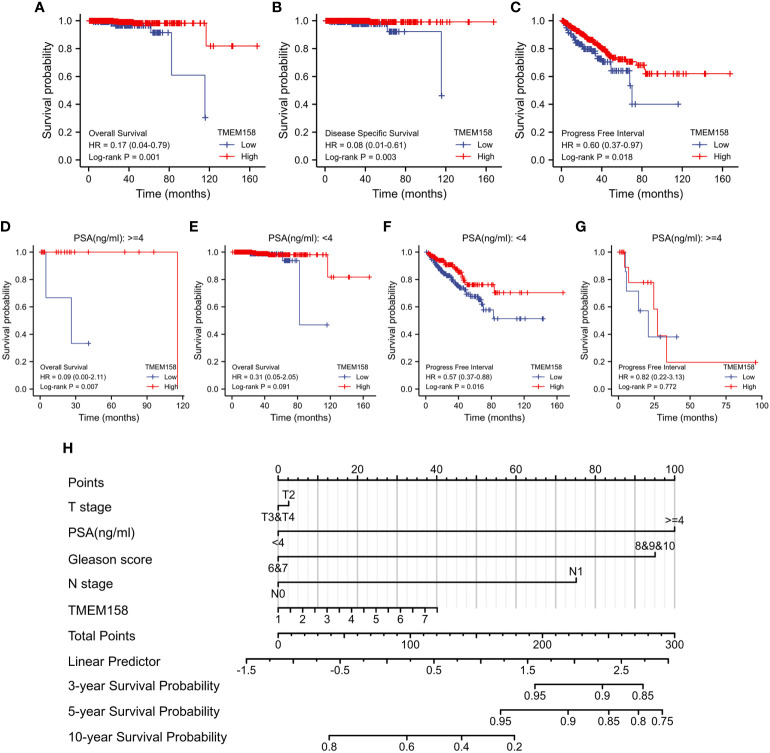
TMEM158 downregulation is associated with survival outcomes. **(A–G)** TMEM158 expression data were extracted from the TCGA-PRAD RNA-seq dataset, and the Kaplan-Meier survival analysis was conducted by stratifying patients using the minimum *p*-value approach (25). **(H)** TMEM158 expression levels and four critical clinicopathological parameters were utilized to generate the nomograph as a survival prediction tool (36). The concordance index is 0.743 (95% CI 0.659-0.826, log-rank test p = 0.0463).

We then determined the prognostic significance of TMEM158 expression for patient survival outcomes. Patients in the TCGA-PRAD dataset were stratified into TMEM158^high^ and TMEM158^low^ groups using the minimum p-value approach. As shown in **Figures 5A–C**, patients in the TMEM158^high^ group had significantly favorite overall and disease-specific survival outcomes and substantially longer progression-free intervals. The advantage of overall survival outcomes in TMEM158^high^ groups was maintained in patients with higher PSA levels (**Figure 5D**) but not in patients with lower PSA levels (**Figure 5E**). Conversely, the advantage of progression-free intervals in TMEM158^high^ groups was only seen in patients with lower PSA levels (**Figure 5F**) but not in patients with higher PSA levels (**Figure 5G**). To apply these data for clinical consultation, we constructed a prognostic calculating table, which integrated clinicopathological information (Tumor stage, PSA level, Gleason score, lymph node invasion) and TMEM158 expression level (**Figure 5H**). This convenient tool can easily calculate predictions for survival outcomes of 3-, 5-, and 10-year ranges after surgery.

### TMEM158 expression significantly correlates with R-Ras and M-Ras gene expression

**Figure 6:**
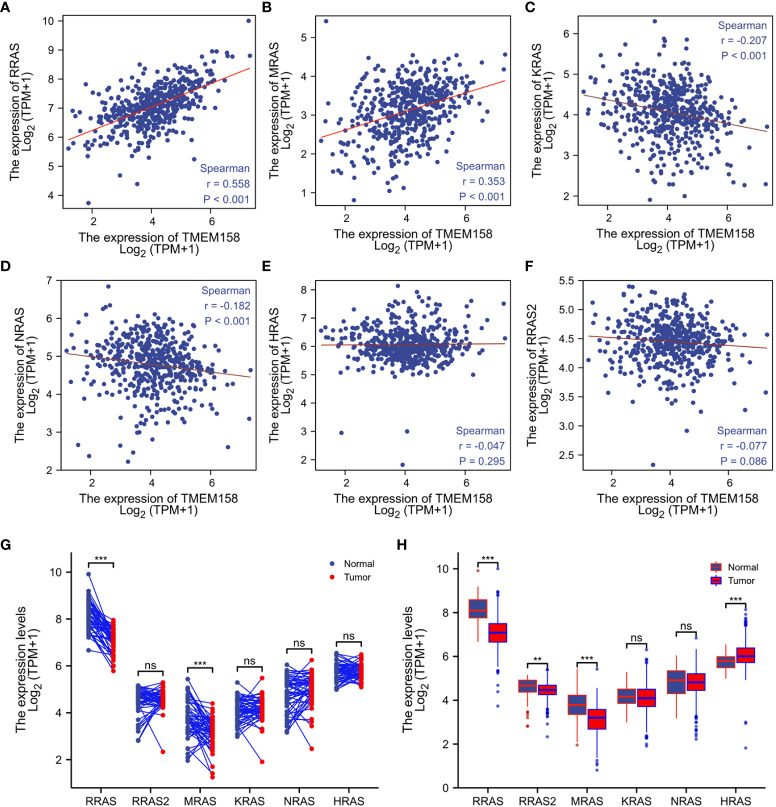
TMEM158 expression is correlated with R-Ras subfamily genes. **(A–F)** Spearman correlation analysis was conducted using the TCGA-PRAD RNA-seq dataset between TMEM158 and Ras family genes. Spearman r > +/- 0.3 was considered as a strong correlation. **(G, H)** Gene expression levels of Ras family genes were extracted from the TCGA-PRAD RNA-seq dataset. Two types of comparison were conducted, case-matched pairwise **(G)** and group cohort comparison. The asterisk indicates a significant difference compared to the normal group (**p < 0.01, ***p < 0.001). ns, no significance.

It was reported that TMEM158 expression was upregulated in response to the Ras pathway activation (5). We then analyzed the correlations of TMEM158 expression with Ras family genes. Our analysis discovered that TMEM158 expression was strongly correlated (Spearman r = 0.558, **Figure 6A**) with R-Ras expression and moderately correlated with M-Ras expression levels (Spearman r = 0.353, **Figure 6B**), but very weakly and negatively correlated with K-Ras and N-Ras expression (**Figures 6C, D**). TMEM158 expression had no significant correlation with H-Ras and R-Ras2 levels (**Figures 6E, F**). R-Ras and M-Ras expression levels were significantly lower in malignant tissues compared to benign prostate tissues pairwise (**Figure 6G**) or in group cohort comparisons (**Figure 6H**). These data suggest that R-Ras signaling might be involved in modulating TMEM158 expression in prostate cancers. However, further investigation is warranted to verify this hypothesis and to confirm the functional significance.

### TMEM158 expression is significantly associated with anti-tumor immune infiltration

**Figure 7:**
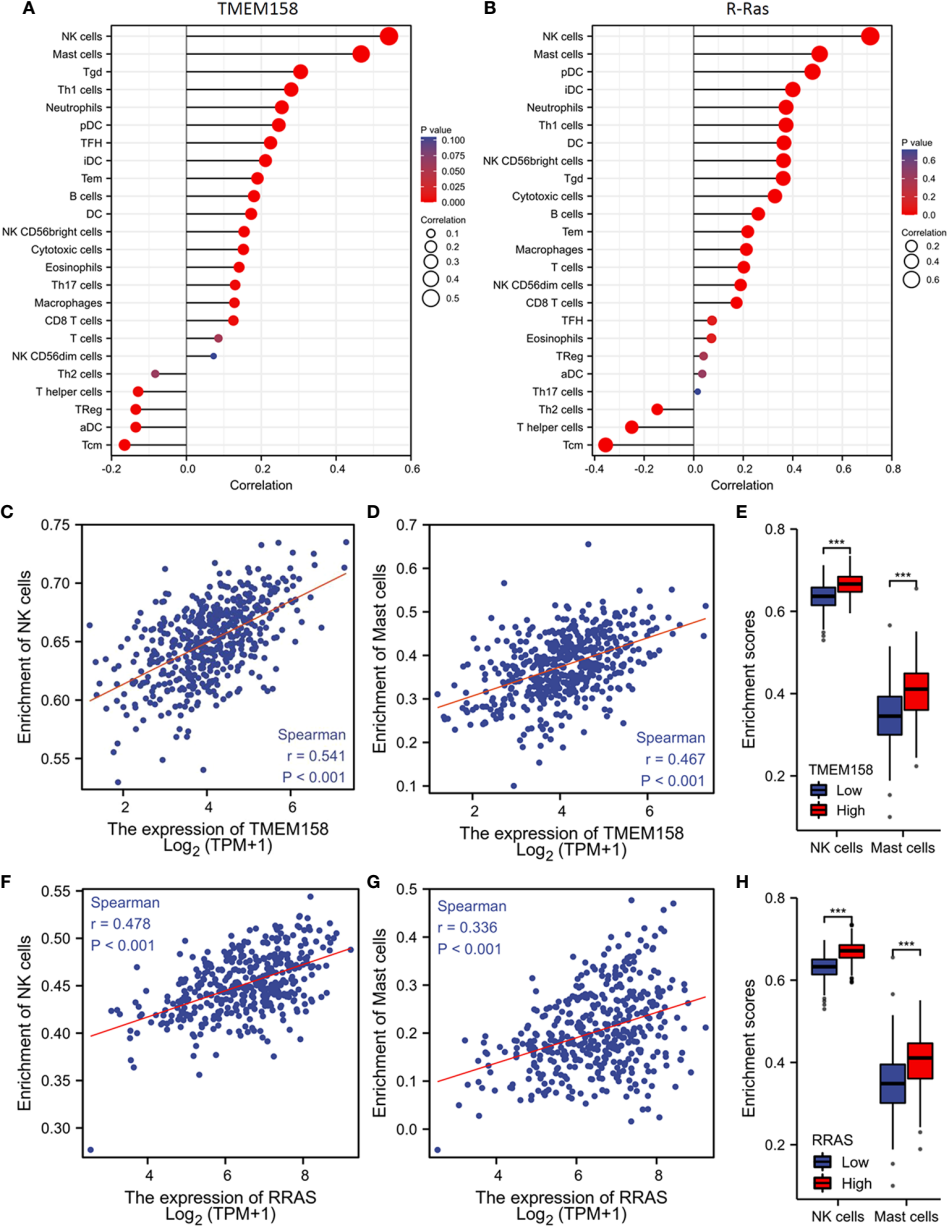
TMEM158 expression is associated with anti-tumor immune filtration. **(A–C)** TMEM158 expression data and immune filtration profiles were extracted from the TCGA-PRAD RNA-seq dataset. The bar graphs were generated using the ssGSEA method. Immune cell category: DC, dendritic cell; aDC, activated DC; iDC, immature DC; pDC, plasmacytoid DC; B cells; CD8 T cells; Cytotoxic cells; Eosinophils; Macrophages; Mast cells; Neutrophils; NK, natural killer cells; NK CD56bright cells; NK CD56dim cells; T cells; T helper cells; Tcm, T central memory; Tem, T effector memory; Tfh, T follicular helper; Tgd, T gamma delta; Th1, T helper-1 cells; Th2 cells; Th17 cells; Treg, T regulatory. **(D–F)** Spearman correlation analysis was conducted using the TCGA-PRAD RNA-seq dataset. **(G–I)** Immune filtration enrichment scores were extracted from the TCGA-PRAD dataset. Patients were divided into groups with TMEM158^low^ or TMEM158^high^ expression levels at the median level. The asterisk indicates a significant difference compared to the benign group (Wilcoxon rank sum test, ***p < 0.001).

Genetic mutation or defects in R-Ras family members were found to cause immune deficiency in mice (37). We then examined the correlations of TMEM158 and R-Ras expression with intra-tumoral immune infiltration profiles. Our analysis revealed that immune infiltration profiles related to the expression levels of TMEM158 and R-Ras genes were very similar, with an overlapped anti-tumor immune filtration (**Figures 7A, B**). TMEM158 expression strongly correlated with the enrichment scores of NK and mast cells (**Figures 7C, D**). Tumors with higher TMEM158 expression consistently showed significantly higher enrichment scores of NK and Mast cell infiltration (**Figure 7E**). Similar results were also observed for R-Ras expression (**Figures 7F–H**). These findings strongly indicate that R-Ras/TMEM158 pathway is associated with anti-tumor immune filtration in prostate cancers.

## Discussion

In this study, we identified the transmembrane protein TMEM158 as a potent prognostic factor for disease-free progression and disease-specific survival in prostate cancer patients. We performed a COX regression analysis on the TCGA-PRAD RNA-seq dataset and found 716 protein-coding genes significantly associated with progression-free interval and disease-specific survival. Among these 716 genes, there were six TMEM family genes; TMEM67, 106C, 126B, 158, 225B, and 272. However, only TMEM158 expression was significantly associated with multiple clinicopathological features, including tumor stage, lymph node invasion, residual tumor after surgery, PSA level, and Gleason score. In patients with aggressive CRPC tumors, TMEM158 expression was negatively correlated with AR gene expression and AR signaling activity but positively correlated with neuroendocrinal features. We also found that TMEM158 expression was suppressed by androgens in prostate cancer cells but increased after androgen removal from the culture media. Our analysis unveiled that TMEM158 expression was strongly associated with R-Ras expression, the R-Ras subfamily members involved in immune modulation (37–39). Interestingly, both TMEM158 and R-Ras were associated with anti-tumor immune filtrations.

TMEM proteins are biological membrane anchoring proteins span the entire lipid bilayers with diverse functionalities (1). Several TMEM family proteins were reported as oncogenic or tumor suppressors in human cancers (1–3). Although TMEM158 was initially proposed as a tumor suppressor gene upregulated during Ras-mediated senescence (5), recent studies suggest that TMEM158 has an oncogenic role in human cancers derived from the colon (6), pancreas (10), ovary (12), glioma (13), and breast (14). In benign tissues, TMEM158 expression is high in the brain, followed by the prostate and spinal cord (**Supplemental Figure S1A**), indicating a significant functional role in these organs. In prostate tissues, TMEM158 expression was mainly found in stromal cells, followed by epithelial, luminal, and basal cells (**Supplemental Figure S1B**). In prostate cancers, TMEM158 expression was significantly lower than in benign tissue compartments, which is different from other cancer types with TMEM158 upregulation. In addition, TMEM158 upregulation was also noticed in other types of human cancers, as reported by others (6, 8, 10–14), including cholangiocarcinoma, esophageal carcinoma, head and neck squamous cell carcinoma, lung adenocarcinoma and squamous cell carcinoma, rectum adenocarcinoma, and stomach adenocarcinoma (**Supplemental Figure S2**). In contrast, renal cell carcinomas, renal chromophobe carcinoma, and uterine corpus endometrial carcinomas showed a significant downregulation of TMEM158 expression compared to their benign counterparts (**Supplemental Figure S2**). Therefore, TMEM158 expression is frequently altered in most of human cancer types, indicating a potential functional role in carcinogenesis or disease progression as an oncogenic factor or tumor suppressor depending on the tumor types.

The R-Ras subfamily consists of three members, R-Ras, R-Ras2, and M-Ras, and they are less studied in human cancers than the classical Ras family proteins (H-Ras, K-Ras, and N-Ras) (37). Recent studies with genetically modified murine models showed that knockout of any R-Ras family genes resulted in reduced immune effector T and B cell populations and defects in innate immune response (38, 40, 41). In addition, it is becoming clear that these R-Ras proteins also regulate cell morphology, adhesion, and migration (42). R-Ras protein has conflicting roles in human cancers, whether oncogenic or tumor suppressive, depending on the cancer types (42). There is a lack in the literature on R-Ras protein function in prostate cancer. Our data showed that R-Ras expression was strongly correlated with TMEM158 expression (Spearman r = 0.558, p < 0.001), and both TMEM158 and R-Ras expression levels were significantly reduced in prostate cancer tissues. Consistent with previous reports that R-Ras is required for immune response, R-Ras and TMEM158 expression were also tightly associated with anti-tumor immune infiltration (NK cell and Mast cell). These results suggest that R-Ras/TMEM158 may play a tumor-suppressive role in prostate cancer, although further investigation is warranted.

AR signal pathway is a critical factor in prostate cancer development and progression, and castration-resistant progression is a major obstacle in prostate cancer management. Our analysis unveiled that TMEM158 expression was inversely correlated with the AR activity score but positively correlated with the NEPC score. Consistently, TMEM158 expression at the mRNA and protein levels were negatively modulated by androgens. These results demonstrated the negative modulation of TMEM158 expression by the AR signal pathway in prostate cancer cells. However, the functional role of TMEM158 downregulation in prostate cancer awaits for further investigation, although the oncogenic roles of TMEM158 overexpression were reported previously through diverse mechanisms, including promoting TGF-β signaling (14), stimulating STAT3 activity (13) or activating PI3K/AKT pathway (10), in other types of human cancers.

## Conclusion

In this study, our analysis revealed that the expression of the membrane protein TMEM158 was significantly downregulated in prostate cancers. TMEM158 downregulation was tightly associated with adverse clinicopathological categories and worse survival outcomes in prostate cancers. In castration-resistant prostate cancers, TMEM158 expression was negatively correlated with AR signaling activity but positively correlated with neuroendocrinal progression. Consistently, androgen treatment suppressed TMEM158 expression in prostate cancer cells, while androgen deprivation led to TMEM158 upregulation. Most importantly, TMEM158 and R-Ras were associated with NK cell and Mast cell infiltration in prostate cancer tissues. Our results suggest that TMEM158 expression is negatively modulated by the AR signal pathway and is a potent prognostic factor for disease progression and patient survival outcomes. The R-Ras/TMEM158 pathway might be involved in anti-tumor immune infiltration.

## Data availability statement

The original contributions presented in the study are included in the article/**Supplementary Material**. Further inquiries can be directed to the corresponding authors.

## Author contributions

BeL and JH designed the study. BiL and BeL analyzed the bioinformatics data. WL, DZ, and BeL conducted the IHC. DZ and BeL drafted the manuscript. All authors contributed to the article and approved the submitted version.

## Funding

The RNA-seq data and patient survival outcomes used in this study are partly based on data generated by the TCGA Research Network (https://www.cancer.gov/tcga). The DoD PCRP PC190026 project partially supported this work to Dr. Benyi Li.

## Conflict of interest

The authors declare that the research was conducted without any commercial or financial relationships construed as a potential conflict of interest.

## Publisher’s note

All claims expressed in this article are solely those of the authors and do not necessarily represent those of their affiliated organizations, or those of the publisher, the editors and the reviewers. Any product that may be evaluated in this article, or claim that may be made by its manufacturer, is not guaranteed or endorsed by the publisher.

## Supplementary material

Supplementary Figure 1
**(A)** TMEM158 expression data in normal organs were extracted from NCBI GDS#422, which was generated using the Affymetrix Human Genome U95 Version 2 Array (43). **(B)** TMEM158 expression data in different prostate cell types were extracted from NCBI GDS#1973. Cell types were separated with magnetic cell sorting (MACS) after pulldown using cell-type specific antibodies, and gene expression analysis at the mRNA level was conducted using the Affymetrix Human Genome U133 Plus 2.0 Array (44).

Supplementary Figure 2 TMEM158 expression in pan-cancer comparison was performed on the GEPIA2 platform with the TCGA datasets. The red box indicates TMEM158 upregulation, and the green box indicates TMEM158 downregulation in tumor tissues. Wilcoxon rank sum test, ** p < 0.01, *** p < 0.001.

Supplementary Table 1 COX aggression analysis of transcriptome with disease-specific survival.

Supplementary Table 2 COX aggression analysis of transcriptome with progression-free interval.

Supplementary Table 3 Prognosis-associated gene list

Supplementary Table 4 TMEM106C expression and clinicopathological parameters in prostate cancers.

Supplementary Table 5 TMEM225B expression and clinicopathological parameters in prostate cancers.

Supplementary Table 6 TMEM272 expression and clinicopathological parameters in prostate cancers.
