# Supplementary figures and images for: TMEM158 expression is negatively regulated by AR signaling and associated with favorite survival outcomes in prostate cancers
Source: Front Oncol. 2022 Nov 1;12:1023455. doi: 10.3389/fonc.2022.1023455 (PMC9663988; doi:10.3389/fonc.2022.1023455)

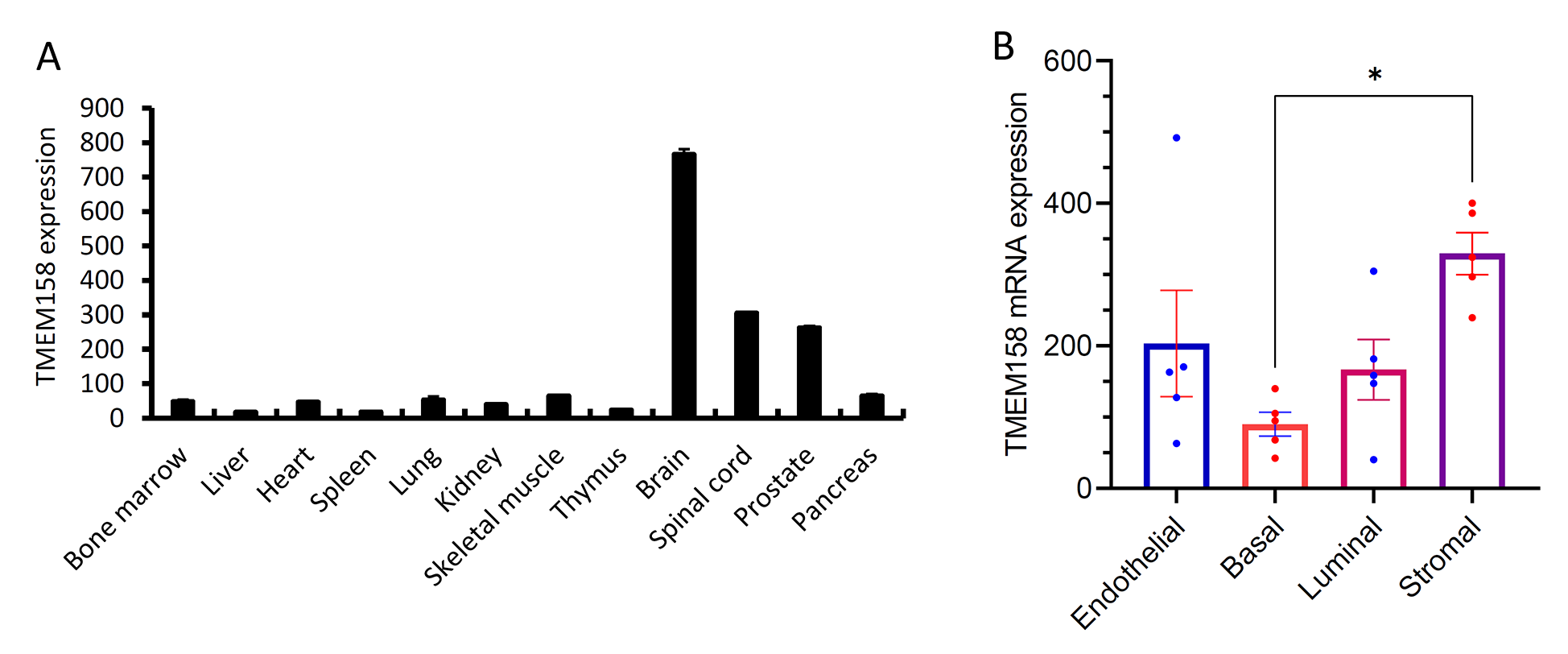

Supplement: Supplementary Figure 1 — (A) TMEM158 expression data in normal organs were extracted from NCBI GDS#422, which was generated using the Affymetrix Human Genome U95 Version 2 Array (43). (B) TMEM158 expression data in different prostate cell types were extracted from NCBI GDS#1973. Cell types were separated with magnetic cell sorting (MACS) after pulldown using cell-type specific antibodies, and gene expression analysis at the mRNA level was conducted using the Affymetrix Human Genome U133 Plus 2.0 Array (44). [file Image_1.tif]

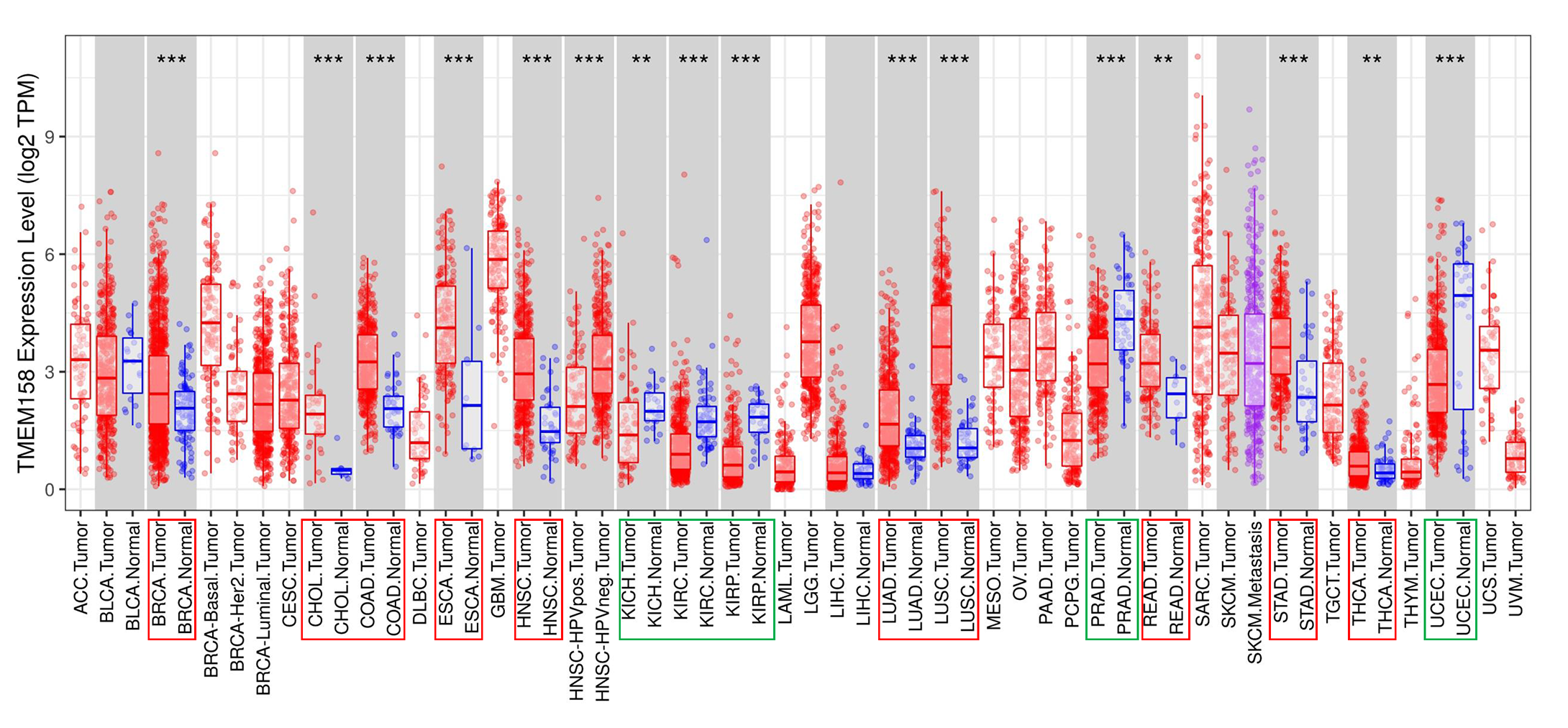

Supplement: Supplementary Figure 2 — TMEM158 expression in pan-cancer comparison was performed on the GEPIA2 platform with the TCGA datasets. The red box indicates TMEM158 upregulation, and the green box indicates TMEM158 downregulation in tumor tissues. Wilcoxon rank sum test, ** p < 0.01, *** p < 0.001. [file Image_2.tif]
